# Therapeutic Approach in the Improvement of Endothelial Dysfunction: The Current State of the Art

**DOI:** 10.1155/2013/252158

**Published:** 2013-01-08

**Authors:** Miroslav Radenković, Marko Stojanović, Tatjana Potpara, Milica Prostran

**Affiliations:** ^1^Department of Pharmacology, Clinical Pharmacology and Toxicology, Medical Faculty, University of Belgrade, P.O. Box 38, 11129 Belgrade, Serbia; ^2^Cardiology Clinic, Clinical Center of Serbia, Medical Faculty, University of Belgrade, 11000 Belgrade, Serbia

## Abstract

The endothelium has a central role in the regulation of blood flow through continuous modulation of vascular tone. This is primarily accomplished by balanced release of endothelial relaxing and contractile factors. The healthy endothelial cells are essential for maintenance of vascular homeostasis involving antioxidant, anti-inflammatory, pro-fibrinolytic, anti-adhesive, or anticoagulant effects. Oppositely, endothelial dysfunction is primarily characterized by impaired regulation of vascular tone as a result of reduced endothelial nitric oxide (NO) synthase activity, lack of cofactors for NO synthesis, attenuated NO release, or increased NO degradation. So far, the pharmacological approach in improving/reversal of endothelial dysfunction was shown to be beneficial in clinical trials that have investigated actions of different cardiovascular drugs. The aim of this paper was to summarize some of the latest clinical findings related to therapeutic possibilities for improving endothelial dysfunction in different pathological conditions. In the majority of presented clinical investigations, the assessment of improvement or reversal of endothelial dysfunction was performed through the flow-mediated dilatation measurement, and in some of those endothelial progenitor cells' count was used for the same purpose. Still, given the fast and continuous development of this field, the evidence acquisition included the MEDLINE data base screening and the selection of articles published between 2010 and 2012.

## 1. Introduction

The endothelium has a central role in the regulation of blood pressure and flow through continuous modulation of vascular tone [1]. This is primarily accomplished by timely and balanced production and release of endothelial relaxing factors, namely, nitric oxide, prostacyclin, or endothelium-derived hyperpolarizing factor [2–4], as well as endothelium-derived contracting autacoids, such as endothelin-1, thromboxane A_2_, angiotensin II, or superoxide anion. The response of different blood vessels to action of various relaxing and contractile vasoactive substances can be partly or entirely endothelium dependent, as well completely endothelium independent [5–8]. Endothelial cells are highly specialized to detect diverse physical, chemical, or mechanical stimuli, such as pulsatile changes in blood flow-induced shear stress, which is pivotal for physiological autoregulation of vascular tone. Apart from the key role in the regulation of vascular tone, healthy endothelial cells continuously adapt to local requirements and are essential for the maintenance of entire vascular homeostasis involving antioxidant, anti-inflammatory, profibrinolytic, and anticoagulant effects. Moreover, in physiological conditions leukocyte adhesion and migration, smooth muscle cell migration and proliferation, secretion of vasoconstrictive factors, and platelet aggregation and adhesion are negatively regulated by undamaged endothelium.

In contrast to previous facts, it is well established that hypertension, smoking, diabetes mellitus, hypercholesterolemia, obesity, or sedentary lifestyle represents major risk factors leading to endothelial dysfunction, atherosclerosis, and other cardiovascular clinical manifestations. Endothelial dysfunction is primarily characterized by impaired regulation of vascular tone as a result of reduced endothelial nitric oxide synthase activity, lack of cofactors for nitric oxide synthesis, attenuated nitric oxide release, or increased nitric oxide degradation. In addition, in this pathological process endothelial cells are directly affected by oxidative stress, increase of endogenous nitric oxide synthesis inhibitors, inflammation, increased adipocytokines, increased release of aldosterone, or depletion of tetrahydrobiopterin [9]. Finally, endothelial dysfunction is also characterized by increased production of endothelium-derived contracting factors, including angiotensin II, endothelins, superoxide anions, and cyclooxygenase-derived prostanoids [10]. In the early stages, endothelial function may be partly maintained by compensatory upregulation of prostacyclin and/or EDHF [11, 12]. Still, the balance between release of endothelial relaxing and contractile factors is shifted toward the vasoconstrictive autacoides. Hence, endothelial dysfunction is expressed in increased interactions with leukocytes, growth and proliferation of smooth muscle cells, prevailing vasoconstriction, impaired coagulation, vascular inflammation, thrombosis, and atherosclerosis [13]. Pharmacological approach in improving/reversal of endothelial dysfunction was shown to be beneficial in clinical trails that have investigated actions of angiotensin converting enzyme inhibitors, angiotensin receptor blockers, statins and other lipid lowering agents, calcium channel blockers, some *β*-receptor blockers, thiazolidinediones, erythropoietin, L-arginine, antioxidants, vitamins, tetrahydrobiopterin, or stimulators of endothelial progenitor cells [9, 14, 15].

The measurement of flow-mediated dilation represents a simple approach to examine vasodilator function in *in vivo* conditions. In particular, flow-mediated dilatation induced by reactive hyperemia has been known to be endothelium dependent, and this can be detected during reactive hyperemia by high-resolution ultrasound in superficial arteries noninvasively [16]. Although many different guidelines are present in the literature, one of the latest comprehensive methodological and physiological guidelines for the assessment of flow-mediated dilation in humans was presented by Thijssen et al. [17]. As outlined in this paper, flow-mediated dilation reflects endothelium-dependent and largely nitric oxide-mediated arterial function and has been used as a surrogate marker of vascular health. This technique has been used to compare groups of subjects and to evaluate the impact of interventions within individuals. Moreover, flow-mediated dilation has become broadly used in clinical studies, in part, because it strongly predicts cardiovascular events in asymptomatic subjects and in patients with established cardiovascular disease. This method is inexpensive, represents a repeatable measure of endothelial function, and can actually provide a fair chance for early assessment of possible cardiovascular outcomes. A blunted vasodilator response to flow-mediated dilation implies a decrease in nitric oxide bioavailability, which may be evoked by different risk factors and pathological conditions that are linked with alterations of signaling pathways in endothelial cells. Thus, improvement of flow-mediated dilation after specific therapeutic approach is translated to the improvement in clinical prognosis. Moreover, the use of flow-mediated dilatation early in drug development programmes provides the possibility to identify not only benefit, but also potential adverse effects on the vessel wall [18].

Endothelial progenitor cells, another powerful marker of endothelial function, are circulating cells with the ability to differentiate into mature endothelium and participate in neoangiogenesis. The circulating endothelial progenitor cells count is now considered to be a predictor of endothelial function and cardiovascular health. Thus, the measurement of endothelial progenitor cells may be a surrogate biologic marker for vascular function and cumulative cardiovascular risk, suggesting further that endothelial injury in the absence of sufficient circulating progenitor cells may unfavorably affect the progression of cardiovascular disease [19]. Furthermore, the assessment of flow-mediated brachial artery reactivity showed a notable relationship between the number of progenitor cells and endothelial function [19].

The aim of this paper was to review and summarize the latest clinical findings related to therapeutic possibilities for improving and restoring endothelial dysfunction in different pathological conditions. In the vast majority of presented clinical investigations the assessment of an improvement or complete reversal of endothelial dysfunction was performed through the flow-mediated dilatation measurement, and in some of those endothelial progenitor cells count was used for the same purpose. Still, given the fast and continuous development of this field, the evidence acquisition involved the MEDLINE data base screening and the selection of articles published between 2010 and 2012. The main findings connected to the improvement of endothelial dysfunction from the reviewed clinical studies were summarized in the Table 1.

## 2. Chronic Heart Failure

The production of nitric oxide has been established to be attenuated in chronic heart failure patients, most probably because of the reduced availability of endothelial nitric oxide synthase and nitric oxide inactivation induced by oxidative stress. Besides, there is an evident decrease of circulating endothelial progenitor cells required for the cellular restoration, too. On the other hand, various pharmacodynamic-unrelated (pleiotropic) actions of statins on patients with chronic heart failure are currently under investigation, given that statins are established to increase vascular nitric oxide production, reduce the production of reactive oxygen species, and promote vasculogenesis.

In randomized, placebo-controlled, and double-blind study reported by Erbs et al. [20], it was of interest to evaluate the pleiotropic effects of rosuvastatin on vascular and tissue regeneration, as well its impact on endothelial function and hemodynamics in chronic heart failure, as a result of ischemic heart disease or dilated cardiomyopathy. Forty-two age-, gender-, and cardiac medication-matched patients clinically stable for at least one month before enrollment were randomized to 12 weeks of oral rosuvastatin (40 mg/day) or placebo. The inclusion and exclusion criteria were in accordance with the clinical status of participants, as well as with the goal of the study, and were appropriately applied. In consideration to endothelial dysfunction, endothelium-dependent flow-mediated dilatation, vascular endothelial growth factor level, oxidized LDL, and the count of circulating progenitor cells were assessed at baseline and after 12 weeks. Rosuvastatin exerted evident antioxidative action. This was substantiated by significant decrease of oxidized LDL levels and lipid peroxidation if compared to placebo group. This was accompanied with notable increase of circulating stem and progenitor cells in rosuvastatin group. Rosuvastatin treatment was also associated with increased plasma vascular endothelial growth factor level. Finally, in rosuvastatin group a significant improvement of flow-mediated dilatation was obtained in contrast to the baseline values in rosuvastatin-treated and the values obtained in placebo group. Although this study provided valuable results considering clinical improvement of chronic heart failure, taking into account only the part considering improvement of endothelial function it can be assumed that rosuvastatin via nonlipid-associated actions notably contributed to neovascularisation and to the enhancement of endothelial function in patients with chronic heart failure.

## 3. Acute Coronary Syndrome

In patients with coronary artery disease angiotensin-converting enzyme (ACE) inhibitors reduce cardiovascular mortality and myocardial infarction rate, most probably by producing beneficial action on functionally damaged endothelial cells and by reversing atherosclerotic process to some point. It is known that decrease in circulating endothelial progenitor cells (mostly characterized by the presence of specific assessment of surface markers, such as CD34 and vascular endothelial growth factor receptor-2) contributes to impaired angiogenesis as well as progression of atherosclerosis, and that patients at risk for coronary artery disease have decreased number of circulating endothelial progenitor cells with impaired activity [19]. On the other hand, vascular endothelial growth factor signaling activity and related angiogenesis plays a protective role in ischemic heart disease and myocardial infarction [34]. Moreover, pharmacotherapy by means of angiotensin II type 1 receptor antagonists or angiotensin-converting enzyme inhibitors has varying effects on progenitor cell levels and function [35].

In the study reported by Cangiano et al. [21] the effect of treatment on endothelial function with perindopril (ACE inhibitor) or valsartan (angiotensin II receptor blocker) at baseline and after 7, 15, and 30 days in patients with non-ST-segment elevation myocardial infarction, and also in normal sex- and age-matched healthy subject controls was investigated. Endothelial apoptosis was determined by cultivating serum samples *in vitro* with human umbilical vein endothelial cells (HUVECs), while endothelial renewal was assessed by necrosis-induced mobilization of CD34+ bone marrow cells. CD34+ cell mobilization is known to be linked with improved prognosis and reduced left ventricular remodeling in postmyocardial infarction patients. At the baseline, postmyocardial infarction patients had around 10 time higher rates of apoptosis if compared with the control group. In the perindopril group the observed parameter significantly declined after 30 days, but not in the group treated with valsartan. Similar results and trends were found for another serum indicator of apoptosis rate, namely, the Bax/Bcl-2 (proapoptotic/antiapoptotic) ratio. CD34+ mobilization was significantly increased in group treated with perindopril, whereas in the valsartan group CD34+ mobilization did not change notably. Positive findings in the perindopril group were associated with an increase in vascular endothelial growth factor, and a reduction in tumor necrosis factor-*α* and its soluble receptors, versus no change in the control. In this paper vascular endothelial growth factor values were strongly correlated with CD34+ cells, and tumor necrosis factor-*α* with the rate of apoptosis. Perindopril also reduced the proapoptotic charge of the serum by increasing the plasma concentration of bradykinin, while angiotensin II concentration was reduced. Thus, it can be suggested that perindopril, unlike valsartan, reduces the proapoptotic effect of serum on the endothelium and increases endothelial renewal in patients with acute coronary syndromes.

## 4. Cardiac Syndrome X

An important clinical entity termed cardiac syndrome X is usually described as angina pectoris with a normal coronary arteriogram [36]. Endothelial dysfunction and inflammation are known to be involved in pathophysiology of this syndrome primarily affecting coronary microvasculature. Treatment options would for example include angiotensin-converting enzyme inhibitors or nitrates. However, the effect of *β*-adrenoceptor antagonists in cardiac syndrome X is still under investigation. Nebivolol belongs to a group of cardioselective *β*-adrenoceptor antagonists having beneficial effects on endothelial function via stimulation of endothelial L-arginine/nitric oxide transduction pathway and augmentation of nitric oxide release [37], which is decreased in plasma of cardiac syndrome X patients.

The action of nebivolol in 38 patients diagnosed with cardiac syndrome X was evaluated in a prospective and randomized clinical trial [22]. The inclusion and the exclusion criteria were adequately defined and appropriately applied. The control group consisted of 18 patients, while the treatment group involved 20 patients receiving nebivolol hydrochloride (5 mg/day *per os*) within the four-week time frame. All ultrasonographic and biochemical analysis were done at baseline and four weeks later. All patients completed the study without any side effects during the followup. Thus, after the nebivolol therapy, there was a significant increase in both brachial artery baseline lumen diameter and lumen diameter after reactive hyperemia, yet without significant change in flow-mediated endothelium-dependent or endothelium-independent dilatation. On the other hand, the levels of serum inflammatory markers indirectly indicating the degree or severity of endothelial function, such as high-sensitivity C-reactive protein, von Willebrand factor, and fibrinogen were significantly decreased, thus leading to a suggestion that nebivolol therapy may have a beneficial effect on endothelial function in patients with cardiac syndrome X.

## 5. Hypertension and Left Ventricular Hypertrophy

Carvedilol belongs to a group of third-generation beta-blockers with nonselective *β*-adrenoceptor, selective *α*(1)-adrenoceptor, and calcium channel blocking activity. This drug was shown to have other beneficial properties on vascular function; including anti-proliferative action on smooth muscle cells or scavenging of free-oxygen species in endothelial cells. In regard to the established association between left ventricular hypertrophy and the presence of endothelial dysfunction [15], possible beneficial action of carvedilol in this setting was not fully investigated. 

In a single-centre, randomized, open, endpoint-blinded, and parallel-group clinical investigation conducted by Xiaozhen et al. [23], the primary goal was to compare the effects of carvedilol on coronary flow reserve in patients with hypertensive left ventricular hypertrophy. However, it was also of interest to investigate endothelium-dependent and endothelium-independent reactivity of the brachial artery through an ultrasound measuring of flow-mediated vasodilatation. In order to further evaluate changes in endothelial function, levels of plasma endothelin-1, nitric oxide, and several other metabolites were monitored and analyzed at baseline and after 6-month therapy. With regards to correctly designed inclusion and exclusion criteria, sixty-three female and male patients, with mean age of 61, were randomly assigned into two groups for treatment with carvedilol (10 mg twice daily) or metoprolol (50 mg). Because of several common beta-receptor blocker-associated side effects, at the end of 6-month therapy, 28 patients remained in the carvedilol group and 29 in the metoprolol group. Antihypertensive action of both drugs was comparable in the final statistical evaluation. Still, taking into account only results regarding the endothelial function, it was found that in carvedilol group endothelium function of the brachial artery was higher than after metoprolol regimen. Namely, in the carvedilol group, flow-mediated arterial dilation increased significantly by approximately 2.5%, as did glyceryl trinitrate-induced endothelium-independent dilatation by approximately 3.3%. Moreover, with the carvedilol treatment, plasma level of endothelin-1 was notably lower, while nitric oxide significantly higher if compared to the metoprolol group or baseline values in both investigated clusters, thus suggesting beneficial and protective effects of carvedilol on endothelial dysfunction in patients with hypertensive left ventricular hypertrophy. This is in accordance with known facts that endothelial dysfunction is usually characterized by decreased release of endothelial relaxing factors, such as nitric oxide, and on the other hand accompanied with increased plasma levels of different endogenous vasoconstrictor substances, such as endothelin-1.

## 6. Coronary Artery Disease and Impaired Glucose Tolerance

It has been established that glitazone-induced activation of PPAR*γ* (peroxisome-proliferator activated receptor-gamma) is positively connected for example with an increase and decrease of adiponectin and TNF-alpha expression, respectively [38]. Pioglitazone belongs to a thiazolidinediones family of antidiabetic drugs having a PPAR*γ* agonistic activity with beneficial effect on insulin sensitivity. PPAR*γ* were first identified as a transcription factor integral to adipocyte differentiation, however, PPAR*γ* are also present in endothelial and vascular smooth muscle cells and they are linked with increased nitric oxide bioavailability, as well with decreased proinflammatory/proatherogenic molecules, primarily through selective regulation of target genes expression [39]. 

In the study of Rizza et al. [24] the hypothesis that modulation of adipocyte function through PPAR*γ* (peroxisome-proliferator activated receptor *γ*) activation may improve insulin sensitivity and adipose/endothelial function was tested. Fifty-four male and female patients with stable coronary artery disease and with a recent-onset impaired glucose tolerance status characterized mainly by insulin resistance but not compromised by glucose toxicity due to hyperglycemia were initially enrolled. In the further course twenty-five coronary artery disease age- and body-mass-index-matched patients with impaired glucose tolerance received either pioglitazone (30 mg/day) or placebo for 12 weeks. Ultrasound assessment of endothelium-dependent and endothelium-independent vasodilator brachial artery reactivity was performed at day one and after 12 weeks. Insulin sensitivity was assessed following the same timeframe. The baseline parameters did not change over the study course in the placebo group. On the other hand, flow-mediated dilatation was significantly improved by approximately 2% at the end of the treatment with pioglitazone. This was also the case with insulin sensitivity connected to significant decrease of tumor necrosis factor-alpha and triglycerides, and increase of high molecular weight adiponectin, as well. The additional analysis suggested that changes in high molecular weight adiponectin levels appeared to be an independent and significant predictor of increased flow-mediated dilatation after pioglitazone treatment. The significance of this result is also connected with the fact that in endothelial cells adiponectin enhances production of nitric oxide, suppresses production of reactive oxygen species, and protects cells from inflammation that results from exposure to high glucose levels or tumor necrosis factor [40].

## 7. Hypertension and Impaired Glucose Tolerance

Treatment-induced reversal of endothelial dysfunction has been confirmed to increase beneficial outcome in hypertensive patients. In consideration to angiotensin-receptor antagonists it has been suggested that telmisartan in serum concentrations obtained in clinical use possesses PPAR*γ* (peroxisome-proliferator activated receptor *γ*) agonistic properties, which is typical for antidiabetic action of glitazones, and improves insulin resistance as well as vascular endothelial dysfunction. This would imply both vascular and metabolic effects of telmisartan, which would be in accordance with recent finding that PPAR*γ* activation inhibited Ang II type 1 receptor expression and protected endothelial function for example by stimulation of endothelial nitric oxide production [41].

In the randomized, double-blind, prospective, and cross-over trial 24 hypertensive patients with impaired glucose tolerance were involved [25]. The patients were 40–75 years old with for at least three months stable parameters including body weight, smoking behavior, physical activity, concomitant medication, and nutritional habits. The exclusion criteria were appropriately defined too. Before an entry into the active period, antihypertensive treatment was switched to amlodipine. Patients were then randomised to telmisartan 80 mg or losartan 50 mg daily for 12 weeks (amlodipine was stopped) in a crossover fashion. At the baseline and after 12 weeks endothelial function testing was performed via ultrasound measuring of flow-mediated vasodilatation, and endothelium-independent, nitroglycerin-mediated vasodilatation, as well. Although both agents showed comparable antihypertensive clinical action, endothelial function improved significantly by telmisartan treatment, but not by losartan administration. Moreover, losartan treatment did not alter flow-mediated vasodilatation recordings if compared to the already low baseline values, indicating initial presence of endothelial dysfunction. The authors also reported that insulin resistance improved significantly after telmisartan only, as did glucose tolerance. This result was independent regarding the improvement of endothelial function. It was also underlined that the effect of telmisartan on insulin resistance and endothelial function is most pronounced in those patients with high insulin resistance and endothelial dysfunction at baseline. 

## 8. Type 2 Diabetes

The prevention and appropriate treatment of type 2 diabetes are today's major issues, given the continuous increase of type 2 diabetes worldwide and consequent vascular complications. Hence, endothelial dysfunction provoked by underlying insulin resistance has been associated with reduced nitric oxide bioavailability, increased production of reactive oxygen species and also with alterations of reparative processes which mediate endothelial regeneration via endothelial progenitor cells [42]. Gliclazide belongs to a second generation of sulfonylurea antidiabetic drugs with additional antioxidant characteristics, most probably connected to free radical scavenging properties of its azabicyclo-octyl ring. This may be also linked with beneficial decrease of low-density lipoprotein oxidation and monocyte adhesion on endothelial cells evoked by diabetic pathological process.

Gliclazide action on possible improvement of flow-mediated dilatation and increase in number of circulating endothelial progenitor cells in type 2 diabetic patients was investigated by Chen et al. [26]. Apart from the inclusion and exclusion criteria, important confounding factors were taken into consideration during the recruitment process. Gliclazide was administered in male and female 33 newly diagnosed type 2 diabetes patients during 12 weeks in a daily dose of 30–90 mg, whereas control group consisted of 25 nondiabetic age- and gender-matched randomly selected subjects. Endothelial function was evaluated by flow-mediated vasodilatation recorded in brachial artery, which was accompanied with measurements of endothelium-independent dilatation induced by sublingual administration nitroglyceride. The number of circulating endothelial progenitor cells co-expressing three characteristic surface antigens (CD45^low^/CD34^+^/VEGFR2^+^) and oxidative stress via malondialdehyde levels and superoxide dismutase activity was also determined. At baseline flow-mediated dilatation, the flow-mediated dilatation/nitroglyceride-mediated dilatation ratio, and the circulating endothelial progenitor cells count were significantly lower in the diabetic group. All quoted parameters were notably improved after 12-week-long gliclazide treatment, yet remained lower then in the control subjects. Still, the percentage increase in flow-mediated dilatation was positively correlated with the percentage increase of endothelial progenitor cells count in the diabetic group. Nitroglyceride-mediated dilatation was comparable in diabetic (before and after the treatment) and the control group. In diabetic patients gliclazide treatment reduced malondialdehyde levels compared to the baseline values. The obtained reduction still remained insufficient to match the values obtained in the control group. Similar trend was observed with the improvement of superoxide dismutase activity. The obtained results were indicative for the assumption that gliclazide could improve endothelial function in diabetes, which may be related to its antioxidative properties.

## 9. Obesity

Obesity has been shown to be one of the most important risk factors for the occurrence or the progression of metabolic syndrome and numerous cardiovascular diseases [43]. Moreover, postprandial hypertriglyceridemia and hyperglycemia are the contributing factors leading to acutely impaired endothelium-dependent vasodilatation and endothelial dysfunction as well. On the other hand, statins are known to improve endothelial function due to lipid-lowering or other pleiotropic actions, involving increased expression of endothelial nitric oxide synthase, negative action of intracellular oxidative stress, or attenuation of proinflammatory pathways [44]. Postprandial hyperlipemia is a phenomenon usually occurring if the dietary sources of fat went above physiological needs and is positively correlated with incidence of obesity, metabolic syndrome, or diabetes. 

In a study including twenty-four obese and drug-naïve male subjects it was of interest to examine if pitavastatin, belonging to a statin group of drugs, can affect postprandial hypertriglyceridemia induced by oral fat loading test and improve endothelial function measured via flow-mediated dilatation methodological procedure [27]. The presence of metabolic syndrome was initially diagnosed in the half of enrolled subjects. During two weeks the participants (age range 29–68; body mass index ≥25 kg/m^2^) with comparable baseline clinical characteristics received either 2 mg/day pitavastatin or placebo. Flow-mediated dilatation was measured before and after four hours of the oral fat load (74 mg of cholesterol per 100 g) in consideration to the fact that serum triglyceride and remnant-like particle cholesterol reach the peak within the same timeframe. Thus, in regard to postprandial response at baseline, after an oral fat load, a notable decrease in endothelium-dependent flow-mediated dilation was observed in all the participants, whereas endothelium-independent nitroglycerin-induced mediated dilation was unaffected. A marked increase of the serum triglyceride level was recorded in all subjects too. Secondly, in the fasting state the pitavastatin group of participants was shown to have an evident increase in the calculated percentages of flow-mediated dilatation and notable decrease in serum triglyceride level, while this was not the case in the placebo group. Finally, in postprandial state the pitavastatin group of subjects was determined to have the complete abolishment of initial decrease of postprandial flow-mediated dilatation at baseline and the further attenuation of the increase in postprandial serum triglyceride level, while oppositely in the placebo group the statistical significance was not observed. The authors also reported that nominal change in postprandial serum triglyceride remained significantly correlated with nominal change in postprandial flow-mediated dilatation among the covariates, including nominal change in postprandial serum low-density lipoprotein cholesterol, triglyceride, and high-density lipoprotein cholesterol. Therefore, it was suggested that the prevention of postprandial endothelial dysfunction by pitavastatin could be at least partly caused by lowering of triglyceride-induced deteriorating actions on endothelial cells. 

## 10. Peripheral Artery Disease

Peripheral artery disease is characterized by partial or complete obstruction of the arteries in the lower limbs. This pathological condition is known to be associated with endothelial dysfunction [45], a damaging vascular process that can be significantly linked with an increased inflammatory status, which also predicts future ischemic events, such as myocardial infarction and stroke [46]. Apart from increased risk for different cardiovascular events, peripheral artery disease is commonly accompanied with the high rate of the quality of life deterioration in affected patients. On the other hand, the preservation of quality of life is for the most part essential to encourage the patients for regular and adequately adjusted physical activity, which is in point of fact a basis of noninvasive treatment of peripheral artery disease—affected population. The angiotensin-converting-enzyme system plays an important role in the pathogenesis and progression of endothelial dysfunction, and ACE inhibitors were shown to be useful for reducing the risk of cardiovascular events in clinical and subclinical peripheral artery disease [47].

The aim of the recent single centre, single-blinded, randomized, and prospective study conducted by Zankl et al. [28] was to analyze the effects of telmisartan (AT_1_-receptor blocker) on absolute walking distance and endothelial function in patients with peripheral artery disease. The authors hypothesized that telmisartan can improve endothelial function, which may further translate into longer maximum walking distance over a period of 12 months. Hence, thirty-six patients between 18 and 80 years of age with documented peripheral artery disease, at least stage Fontaine IIa, were assigned to a telmisartan or placebo group for 12 months, which included four visits (baseline visit, after four weeks, and finally after six and twelve months). Telmisartan was initially administered in a standard dose of 40 mg/day and up-titrated after four weeks to 80 mg/day, if clinically necessary. Absolute walking distance was measured at baseline visit and after six and 12 months by standard treadmill exercise test. In order to measure local endothelial release of nitric oxide, vascular ultrasound scans of the right brachial artery blood flow were recorded following standard flow-mediated vasodilatation protocol with ischemia and reactive hyperemia episodes. After 12 months, maximum walking distance increased by 26% (from 132 m at baseline to 191 m) in the telmisartan group, whereas in the placebo group it was comparable to baseline. At the same time flow-mediated vasodilatation was augmented by 40% (from 0.06 mm at baseline to 0.08 mm) in the telmisartan group, while it actually deteriorated in the placebo group (from 0.05 mm to 0.04 mm). Even though a questionnaire-estimated disease-related quality of life scores remained unchanged in the telmisartan group, placebo treatment was associated with a marked decline by 30%. In this pilot study there was a clear beneficial effect on brachial artery endothelial function in patients with peripheral artery disease subjected to treatment with telmisartan. The improvement of endothelial function was also shown to be translated into clinical advantages from which patients would benefit, that is to say walking capacity and the quality of life.

## 11. Behçet's Disease

Behçet's disease is rare multisystem disorder, which affects mostly young adults and it is described as a chronic immune-inflammatory vasculitis with perivascular infiltration affecting vessels of various sizes [48]. Vascular invasion by activated leucocytes and consequential production of free-oxygen species provides an excellent ground for the pathological activation and final dysfunction of endothelial cells. Although recurrent oral and genital ulcers and uveitis are the clinical trial of the disease, the vascular system involvement represents the main cause of mortality. The treatment of Behçet's disease is usually individualized and adjusted to the specific clinical findings in each patient, yet mostly symptomatic and empirical.

The effect of atorvastatin (HMG-CoA reductase inhibitor) and lisinopril (ACE inhibitor) on endothelium dysfunction was investigated in patients with Behçet's disease [29]. The undertaken investigation was designed as a prospective, randomized, double-blind, and placebo-controlled study enrolling 92 male and female patients diagnosed with Behçet's disease, and with ultrasonographically documented endothelial dysfunction as well. Patients clearly presenting pathological conditions or other risk factors known to contribute to occurrence and/or progress of endothelial dysfunction were initially excluded. Age, body mass index, systolic blood pressure, diastolic blood pressure, total cholesterol, HDL cholesterol, LDL cholesterol, fibrinogen, and erythrocyte sedimentation rate were comparable between the participants. Endothelial dysfunction was evaluated by brachial artery flow-mediated dilatation at baseline and after three months of atorvastatin (20 mg/day), lisinopril (10 mg/day), or placebo regimen. The obtained recordings were expressed as the percentage change in the brachial artery diameter from baseline to the subsequent reactive hyperemia. Thus, a notable improvement in endothelium-dependent flow-mediated dilatation by 7.8% was observed in atorvastatin group. This was also the case in patients with Behçet's disease initially assigned to lisinopril administration, where baseline results improved by 6.4%. Still, only partial enhancement of 0.8% was observed in placebo group. Apart from the previous findings, it was shown that reduced LDL level before and after three months was notably linked with an improvement of flow-mediated dilatation in atorvastatin group. In consideration to endothelium-independent vasodilatation provoked by sublingual nitroglycerine, there was a similar trend in improvement of dilatation in participants receiving lisinopril and atorvastatin. The obtained results were indicative for the assumption that atorvastatin and lisinopril improved endothelial functions in Behçet's disease patients, thus augmenting therapeutic possibilities for better treatment of this pathological condition.

## 12. Polycystic Ovary Syndrome

Polycystic ovary syndrome, representing a common endocrine disorder in women of reproductive age, has been established to have a positive correlation with increased risk of gestational diabetes, type 2 diabetes and other associated cardiovascular pathological conditions [49]. As a marker of pathological vascular damage, endothelial dysfunction was associated with polycystic ovary syndrome, as well. One of the possible reasons for endothelial dysfunction in this pathological condition could be related to androgens' proinflammatory and oxidative stress-related actions [50]. One of the accepted approaches for polycystic ovary syndrome treatment involves spironolactone, an androgen and mineralocorticoid receptor blocking drug, especially in patients with hirsutism or other androgen excess-associated clinical aberrations. 

The assumption that treatment with spironolactone might reverse endothelial dysfunction in nonobese polycystic ovary syndrome patients was recently examined by Bajuk Studen et al. [30]. Thirty nonobese patients with polycystic ovary syndrome were compared with twenty healthy body mass index-matched women having a regular menstrual cycles and no clinical androgenism or biochemical hyperandrogenemia. All the inclusion and the exclusion criteria were appropriately defined and applied. Polycystic ovary syndrome patients were given spironolactone 100 mg daily in 21-day long intervals followed by a 7-day pause regardless of menstrual bleeding, for 6 months. In the hemodynamic examination an endothelium-dependent flow-mediated dilatation and endothelium-independent glyceryl trinitrate-induced dilatation of the brachial artery were studied. At baseline flow-mediated dilatation was significantly lower in polycystic ovary syndrome group comparing to healthy women (9.0 versus 10.2%, resp.). This was also the case with glyceryl trinitrate-induced dilatation (polycystic ovary syndrome (21.3%) versus control (26.6%)). After six months of spironolactone administration, all participants of the study were reevaluated and the flow-mediated dilatation was found to be notably improved in polycystic ovary syndrome group, while it remained unchanged in controls. Furthermore, the obtained percentages for flow-mediated dilatation after six months were comparable between treated and untreated women (8.3 versus 9.4%, resp.). Endothelium-independent glyceryl trinitrate-induced dilatation of the brachial artery remained unchanged. It was proposed that in this study, six months of spironolactone treatment reversed endothelial dysfunction in nonobese polycystic ovary syndrome patients primarily through its antiandrogenic effects and partly via aldosterone antagonism.

## 13. Subclinical Hypothyroidism

Subclinical hypothyroidism is medical condition, which involves normal serum free T4 (FT4) and free T3 (FT3) levels, while serum thyroid-stimulating hormone (TSH) levels are increased [51]. This endocrine pathological condition is considered to be one of the risk factors for atherosclerotic cardiovascular disease, and has been associated with dysfunction of endothelial cells, as well. The increase in cardiovascular risk, most clearly associated with hyper- and hypothyroidism, is due not only to alterations in lipid profile, but also to hemodynamic changes, endothelial dysfunction, coagulation disturbances, hormonal and metabolic changes, and changes in measurable factors such as homocysteine and C-reactive protein, which are known to increase risk for atherosclerotic disease [52]. Although it has been shown that thyroid hormone replacement therapy improves endothelial function in patients with hypothyroidism [53], the potential beneficial effects of L-thyroxin on damaged endothelium are still under investigation.

In the prospective study reported by Alibaz Oner et al. [31] it was of interest to evaluate endothelial functions and the effect of L-thyroxin therapy on endothelial functions in young adults without any cardiovascular disease concomitantly diagnosed with subclinical hypothyroidism. Twenty-seven patients with newly diagnosed subclinical hypothyroidism were prospectively enrolled. They were age- and sex-matched with twenty-two healthy subjects, representing a control group. The exclusion criteria were adequately applied. TSH, FT3, and FT4 levels were determined in all participants at baseline. Thus, TSH levels were significantly higher, while FT4 levels were notably lower in subclinical hypothyroidism group. Baseline brachial artery ultrasonographic parameters of endothelium-dependent flow-mediated dilatation where significantly lower in subjects with subclinical hypothyroidism compared to healthy controls, while endothelium-independent nitrate-induced dilatation remained comparable between examined groups. Oral L-thyroxin (L-T4) was administered at daily dose of 0.5 *μ*g/kg and the patients were followed up and repeatedly tested in regard to thyroid function until euthyroidism was restored. Brachial ultrasonography was performed again 4–6 weeks after the restoration of euthyroidism with L-T4 therapy. Thus, baseline diameter of brachial artery, flow-mediated dilatation value, and nitrate-induced dilatation all significantly improved after the L-T4 treatment in subclinical hypothyroidism patients if compared to the baseline values. This was accompanied with significant increase of FT4 levels and decrease of TSH and total cholesterol levels. This investigation showed that L-T4 treatment in subclinical hypothyroidism patients was associated with significant improvement of endothelial dysfunction. Moreover, the improvement of nitrate-induced endothelium-independent dilatation suggests positive action of L-T4 on vascular smooth muscle cells too. 

## 14. Ankylosing Spondylitis

Ankylosing spondylitis is defined as a chronic inflammatory rheumatic disease affecting sacroiliac joints and spine, which is associated with an increased cardiovascular risk, especially atherosclerosis and coronary artery disease. It is considered that the inflammatory component underlying ankylosing spondylitis pathological process contributes at least in one part to the overall cardiovascular risk and specifically to endothelial dysfunction, most probably as a consequence of increased proinflammatory cytokines levels, for example tumor necrosis factor alpha (TNF-*α*), which in turn negatively regulates endothelial function with resulting decrease of endogenous nitric oxide bioavailability [54]. Thus, as previously outlined, targeting of adhesion molecules, chemokines, and angiogenesis by administering nonspecific immunosuppressive drugs as well as monoclonal antibodies or small molecular compounds inhibiting the action of a single mediator may control inflammation and prevent tissue destruction [55].

The hypothesis that chronic inflammation in ankylosing spondylitis is associated with endothelial dysfunction despite treatment with conventional disease modifying antirheumatic drugs, which can be reversed with intravenous (i.v.) infusion of infliximab (anti-TNF-*α* agent) was tested in the prospective, uncontrolled, open-label, pilot study by Syngle et al. [32]. The study participants were twelve anti-TNF-naïve ankylosing spondylitis patients with high disease activity, lacking an adequate response to conventional disease modifying antirheumatic drugs. The inclusion criteria were well applied and only patients on stable dose of disease modifying antirheumatic drugs (diclofenac or indomethacin + sulfasalazine) for at least 6 months were included in the study. These patients were administered infliximab 5 mg/kg as a single i.v. infusion. The exclusion criteria were presented in detail and were in accordance with overall design and aim of the study. In consideration to the endothelial function, serum nitrite (nitric oxide surrogate metabolite marker) concentration and flow-mediated, as well as nitroglycerin-induced dilatation of brachial artery were evaluated at baseline and 12 weeks after single-dose treatment with infliximab. Thus, flow-mediated dilation was significantly improved by approximately 17% after 12 weeks, whereas endothelium-independent nitroglycerin-induced dilatation and baseline brachial artery diameter remained unchanged. Twelve weeks after single-dose treatment with infliximab serum nitrite concentration was notably decreased (approximately 4 *μ*mol/L) if compare to the baseline. Infliximab also reduced markers of inflammatory disease activity (erythrocyte sedimentation rate and C-reactive protein levels). This all was indicative for the assumption that the protective effect of infliximab was preserved for at least 12 weeks after initial infusion. The authors suggested that in ankylosing spondylitis, endothelial dysfunction is a part of the disease process and infliximab improves both endothelial dysfunction and inflammatory disease activity, thus providing a promising approach to manage spondylitis and its atherosclerotic complications.

## 15. Chronic Hemodialysis

One of the principal origins of cardiovascular morbidity in patients undergoing hemodialysis is associated with the occurrence and the progression of atherosclerotic pathological process with ischemic heart disease and myocardial infarction as resulting consequences. The reasons for end-stage renal disease patients having signs of endothelial dysfunction are multifactorial, including increased oxidative stress, hyperhomocysteinemia, dyslipidemia, hyperglycemia, hypertension, or retention of L-arginine [56]. On the other hand, statins are confirmed to reduce risk of coronary events through decrease of plasma lipid levels and improving endothelial dysfunction via upregulation of endothelial nitric oxide and antioxidant activity, as well.

The effects of simvastatin on lipids, flow-mediated endothelium-dependent and nitroglycerin-induced endothelium-independent dilatation, and markers of oxidant stress and atherosclerosis were investigated in 37 patients on hemodialysis (lasting approximately 4 years) with or without mild hyperlipidemia, as well without lipid-lowering drugs for at least six weeks before the study beginning, and also without any acute coronary events for at least three months prior to the study [33]. The participants were age- and gender-matched and based on the plasma LDL levels they were enrolled into (a) 5 mg/day simvastatin group (*n* = 14), (b) 10 mg/day simvastatin group (*n* = 14), and (c) the control group without prescription (*n* = 9) for 16-week interval. All groups were matched in consideration to associated pathological conditions and regular use of medications that can affect endothelium-mediated physiological functions. Hence, the endothelium-dependent flow-mediated dilatation in simvastatin groups notably increased at week 1 and 16, while nitroglycerin-induced endothelium-independent dilatation remained unchanged over the study course. Markers associated with endothelial dysfunction, such as oxidized LDL, soluble vascular cell adhesion molecule 1, and 8-epi-prostaglandin F2, significantly decreased after 16 weeks in both statin groups, while on the other hand plasma nitrite/nitrate increased, thus indicating increased nitric oxide bioavailability. It was concluded that simvastatin improved the impaired endothelial function of dialysis patients by decreasing oxidized LDL, improving the lipid profile and, at least in part, enhancing nitric oxide bioavailability.

## 16. Conclusion

The knowledge about the risk factors, molecular mechanisms and related intracellular signaling pathways, and nonpharmacological and pharmacological treatment options regarding endothelial dysfunction is still accumulating. Although this paper covered some of the recent findings obtained with the clinical administration of primarily conventional drugs, such as statins, angiotensin-converting enzyme inhibitors, angiotensin receptor blockers, beta-adrenoceptor antagonists or oral antidiabetic drugs, quite a few articles were published involving the investigation of different dietary supplements, naturally-occurring compounds, food ingredients or related substances on endothelial function. Thus, over the last two years positive correlations considering the improvement of endothelial function in regard to flow-mediated dilation were confirmed, or newly established after enteral or parenteral use of polyphenols (resveratrol, hesperidin), folic acid, *N* ^1^-methylnicotinamide, antioxidant agents (vitamin C, E, *α*-lipoic acid, co-enzyme Q10), omega-3-polyunsaturated fatty acids, then after consumption of flavanol-rich cocoa, walnut-enriched *ad libitum* diet or flavonoid- and nitrates-rich food, as well after induction of tetrahydrobiopterin synthesis [57–68]. Taken together, clinical investigations aiming the improvement and/or complete reversal of endothelial dysfunction are constantly being undertaken, thus giving the additional input to adequate prevention, diagnosis, and nonpharmacological and pharmacological treatment of different cardiovascular diseases and pathological conditions associated with later cardiovascular pathology. 

## Figures and Tables

**Table 1:** The main findings connected to the improvement of endothelial dysfunction from the reviewed clinical studies.

Pathological condition	Drug	Main effects	Corresponding reference
Chronic heart failure	Rosuvastatin	Oxidized LDL ↓ Lipid peroxidation ↓ Stem and progenitor cells ↑ FMD ↑	Erbs et al. [20]

Acute coronary syndrome	Perindopril	Apoptosis ↓ CD34+ mobilization ↑ VEGF ↑ TNF-*α*↓ Bradykinin ↑	Cangiano et al. [21]

Cardiac syndrome X	Nebivolol	FMD ↔ High-sensitivity C-reactive protein ↓ von Willebrand factor ↓ Fibrinogen ↓	Kayaalti et al. [22]

Hypertensive left ventricular hypertrophy	Carvedilol	FMD ↑ Endothelin-1 ↓ Nitric oxide ↑	Xiaozhen et al. [23]

Coronary artery disease and impaired glucose tolerance	Pioglitazone	FMD ↑ TNF-*α*↓ Triglycerides ↓ HMW adiponectin ↑	Rizza et al. [24]

Hypertension and impaired glucose tolerance	Telmisartan	FMD ↑ Insulin resistance ↓ Glucose tolerance ↑	Perl et al. [25]

Type 2 diabetes	Gliclazide	FMD ↑ Endothelial progenitor cells ↑	Chen et al. [26]

Obesity	Pitavastatin	FMD ↑ Triglycerides ↓	Nagashima and Endo [27]

Peripheral artery disease	Telmisartan	Maximum walking distance ↑ FMD ↑	Zankl et al. [28]

Behçet's disease	Atorvastatin Lisinopril	FMD ↑	Inanc et al. [29]

Polycystic ovary syndrome	Spironolactone	FMD ↑	Bajuk Studen et al. [30]

Subclinical hypothyroidism	L-thyroxin	FMD ↑ FT4 ↑ TSH ↓ Total cholesterol ↓	Alibaz Oner et al. [31]

Ankylosing spondylitis	Infliximab	FMD ↑ Serum nitrite ↓ Erythrocyte sedimentation rate ↓ C-reactive protein ↓	Syngle et al. [32]

Chronic hemodialysis	Simvastatin	FMD ↑ Oxidized LDL ↓ VCAM-1 ↓ 8-epi-PG F2 ↓ Nitric oxide bioavailability ↑	Kishimoto et al. [33]

FMD: flow-mediated dilatation; VEGF: vascular endothelial growth factor; TNF-*α*: tumor necrosis factor-*α*; TSH: thyroid-stimulating hormone; VCAM-1: vascular cell adhesion molecule 1.
